# Adjustment to acute or early HIV-1 infection diagnosis to prompt linkage to care and ART initiation: qualitative insights from coastal Kenya

**DOI:** 10.1080/13548506.2018.1549736

**Published:** 2018-11-23

**Authors:** EM Van Der Elst, B Kombo, P Mugo, A Thiong’o, J Kanungi, E Wahome, O Chirro, SM Graham, D Operario, EJ Sanders

**Affiliations:** a Kenya Medical Research Institute-Wellcome Trust Research Programme, Kilifi, Kenya; b Department of Global Health, Academic Medical Centre, University of Amsterdam, Amsterdam, Netherlands; c Departments of Medicine, Global Health, and Epidemiology, University of Washington, Seattle, WA, USA; d Department of Behaviour and Social Sciences, Brown University School of Public Health, RI, USA; e Centre for Topical Medicine and Global Health, Nuffield Department of Medicine, University of Oxford, Oxford, UK

**Keywords:** Acute and early HIV-1 infection, early ART initiation, behavioral adjustment, linkage to care, Kenya

## Abstract

Diagnosing and treating patients with acute or early HIV-1 infection (AEHI) is an important strategy to prevent HIV-1 transmission. We used qualitative methods to understand factors that facilitate adjustment to AEHI diagnosis, prompt linkage to care and initiation of antiretroviral treatment (ART). Twenty-three AEHI patients (12 women, 11 men) included 18 participants identified at health facilities, and 5 participants identified in a sex worker cohort. Of these, 17 participants (9 women, 8 men) participated in qualitative interviews about their AEHI status 2 weeks after diagnosis. Thirteen participants (7 women, 6 men) returned for a second interview 12 weeks after diagnosis. Interviews explored participants’ experiences at the time of and following their diagnosis, and examined perceptions about ART initiation and behavior change recommendations, including disclosure and partner notification. A grounded theory framework was used for analysis, eliciting three important needs that should be addressed for AEHI patients: 1) the need to better understand AEHI and accept one’s status; 2) the need to develop healthy strategies and adjust to the reality of AEHI status; and 3) the need to protect self and others through ART initiation, adherence, safer sex, and disclosure. A preliminary conceptual framework to guide further intervention and research with AEHI populations is proposed.

## Introduction

Early antiretroviral therapy (ART) initiation is one of the most effective HIV prevention interventions to date. People with acute or early HIV-1 infection (AEHI) are highly infectious, but prompt initiation of and adherence to ART can reduce transmission by up to 96% (Ambrosioni et al., 2012; Cohen, Shaw, McMichael, & Haynes, 2011). However, if left unrecognized and untreated, both acute and early infection periods substantially heighten the likelihood for ongoing HIV-1 transmission (Fiebig et al., 2003; Smith et al., 2013) and can lead to impaired immunologic function and increased HIV reservoir size in infected people (Cuadros, Awad, & Abu-Raddad, 2013; Jones et al., 2014). Little is known about the experiences of initiating ART among individuals diagnosed with AEHI.

The phase of acute HIV-1 infection comprises the first 2 to 3 weeks after HIV-1 acquisition and is defined in this study as having a positive HIV nucleic acid amplification or p24 antigen test accompanied by either negative or discordant rapid antibody test results (Powers et al., 2007). By contrast, early HIV-1 infection, inclusive of the acute phase, represents an approximately 6-month period of increased transmission risk (Rutstein et al., 2017). Both are critical window periods during which condomless sex acts can lead to a high reproductive rate of HIV in the population (Powers et al., 2011; Zhang et al., 2012). Detection of AEHI in low- and middle-income countries, however, is rather uncommon (Miller, Rosenberg, Rutstein, & Powers, 2010).

In the Kenyan coast where HIV is endemic, studies indicate that acute HIV-1 can be as common as malaria in young febrile adults (Sanders et al., 2014, 2011). Targeted AEHI testing can be effective at diagnosing AEHI patients (Sanders et al., 2015), and represents an opportunity for early treatment initiation, which if implemented can produce individual clinical benefits (Ananworanich, Dube and Chomont 2015; Krebs & Ananworanich, 2016), as well as public health benefits such as reduced risk for sexual and perinatal HIV transmission (Martin et al., 2017; Sereti et al., 2017). Since 2016, WHO guidelines recommend treatment of all HIV-positive individuals regardless of CD4 cell count (WHO, 2018).

This paper explores the factors that can enable or obstruct ART initiation and risk reduction recommendations among patients diagnosed with AEHI in coastal Kenya. The psychosocial challenges associated with receiving an AEHI diagnosis, such as emotional distress and coping difficulties, can potentially affect ART uptake and compliance with behavior change recommendations, such as sexual risk reduction and partner notification (Remien et al., 2009). Therefore, findings from this research can provide insight into strategies for improving clinical counseling and health promotion for newly diagnosed AEHI patients.

## Methods

### Participants

The study was conducted in Kilifi County, Kenya, between 2013 and 2016. Participants were recruited through two means. First, AEHI patients were identified when they sought healthcare from health facilities where providers screened for AEHI symptoms. Individuals with rapid HIV negative or discordant test results were offered evaluation with a p24 antigen or Xpert® test, and if tested positive, diagnosed with AEHI (Sanders et al., 2017, 2014). Second, participants from an ongoing at-risk male sex worker cohort were eligible for participation when their HIV-1 infection was diagnosed within 1–3 months of their last HIV-1 negative cohort visit. At the point of diagnosis, providers discussed with AEHI patients the clinical factors associated with AEHI status, high risk of transmission to sexual partners, and the advantages of immediate treatment. During 2013 and 2014, AEHI patients were invited to initiate HIV clinical care services, including risk reduction counseling and ART according to Kenyan guidelines (NASCOP, 2014) free of charge at the Kenya Medical Research Institute (KEMRI). As from 2016, AEHI patients were offered HIV partner notification services as per Kenya’s renewed ART guidelines (Ministry of Health, National AIDS & STI Control Programme, 2016).

### Interview procedures

AEHI-diagnosed index patients were invited to participate in two in-depth individual interviews: the first interview within 2 weeks following AEHI diagnosis and the second interview approximately 3 months following AEHI diagnosis. The interview schedule was designed to capture participants’ perceptions about their diagnosis and treatment experiences during the period in which they were most infectious but aware of their HIV status. Topics for the first interview included participants’ prior HIV testing history, sexual behaviours and partnerships, knowledge about AEHI-related health issues, infectiousness, and ART. The second interview explored participants’ experiences following the first interview, such as changes in sexual behaviours, disclosure of HIV-1 status to partners, use of staff assistance with disclosure to partners, and ART initiation and experiences.

In-depth interviews were conducted in Kiswahili by research team members, who followed semi-structured interview guides. Interviews were conducted in private counseling rooms. The first interview lasted approximately 75 minutes, while the second interview took approximately 90 minutes, as additional time was needed to reflect on topics discussed during the first interview. At the end of each interview, the interviewer provided specific education and counseling to correct misinformation and misunderstandings that emerged during the interviews. Participants received 300 Kenyan shillings (approximately $3USD) following each interview to compensate for travel and time.

Ethical approval was obtained from KEMRI’s Scientific & Ethics Review Unit and the University of Oxford. All participants provided written informed consent for both interviews. Specific protocols for safety measures to protect participants’ privacy and confidentiality, as well as for provision of mental health support and counseling were in place. The study team made every effort to link participants to care and additional services as needed.

### Analysis

Interviews were audiotaped, transcribed, translated into English, and entered into NVivo 10 software. Personal identifying details were omitted. Following a grounded theory approach (Charmaz, 2006), we sought to build a framework for understanding factors that enable or obstruct patient’s willingness to initiate ART and change behaviours. The initial coding inductively generated as many ideas as possible from the data. Extensive case-based and conceptual memos were written that contained interviewers’ impressions about patients’ experiences, which guided follow-up questions for second-round interviews. Use of the constant comparative method allowed this analytic process to produce not just a description of concepts but a preliminary framework explaining relationships between concepts. Researchers met regularly to discuss emerging concepts and interpretations.

## Results

During the recruitment period, 23 patients with AEHI (12 women -including 2 pregnant women-, 11 men; 18 health facility patients, 5 cohort participants) were diagnosed and invited to participate in qualitative interviews (Table 1). The median age at diagnosis was 28 years for women and 26 years for men. Nineteen patients registered for HIV care at KEMRI, of whom 17 participated in this study while two refused. All 17 completed the first interview (9 women; 8 men) and 13 (7 women; 6 men) completed the second interview. Of the 5 (2 women; 3 men) who did not complete the second interview, two female patients opted to seek HIV care elsewhere because they preferred another facility, no follow-up HIV care information was available on the three male patients.

**Table 1.** Outcomes of care linkage, ART initiation, and partner HIV status of 23 participants with acute or early HIV infection (AEHI) targeted for qualitative interviews, Coastal Kenya, 2013–2016.

No.	Sex	Age	Patient identified at	Linkage outcome	Partner HIV status	No. of qualitative interviews
1	F	22	HF^1^	LFU^2^ at diagnosis	Unknown	0
2	M	23	HF	Enrolled	HIV-positive	2
3	F	28	HF	Enrolled	HIV-negative	2
4	F	25	HF	Enrolled	HIV-negative	2
5	F	26	HF	LFU following enrolment	Unknown	1
6	F	24	HF	Enrolled	HIV-positive	2
7	M	26	HF	LFU at diagnosis	Unknown	0
8	M	21	HF	Enrolled	HIV-positive	1
9	F	28	HF	Enrolled	HIV-positive	2
10	M	33	HF	LFU following enrolment	Unknown	0
11	M	26	HF	LFU at diagnosis	Unknown	0
12	M	29	HF	Enrolled	HIV-positive	2
13	M	22	Cohort	Enrolled	Unknown	1
14	M	29	Cohort	Enrolled	HIV-negative	2
15	M	29	Cohort	Enrolled	HIV-positive	2
16	M	24	Cohort	Enrolled	Unknown	2
17	M	24	Cohort	Enrolled	Unknown	2
18	F	29	HF	LFU following enrolment	Unknown	0
19	F	32	HF	Enrolled	HIV-positive	2
20	F	29	HF	Enrolled	HIV-positive	2
21	F	28	HF	Enrolled	HIV-positive	2
22	F	23	HF	Enrolled	HIV-negative	1
23	F	27	HF	LFU at diagnosis	Unknown	0

^1^HF = Health Facility ^2^LFU = Loss to Follow Up

Within approximately one month of registration, 13 partners from 17 participating index patients (one refused partner notification services) were successfully contacted by the research staff and invited for either couple or individual HIV testing and counseling, according to the partner’s preferences. Of the 13 partners tested, 9 partners (4 women; 5 men) tested seropositive, of whom 7 partners (2 women; 5 men) were newly diagnosed.

### Overview of key concepts

Based on our analysis of qualitative data, we identified three key issues characterizing the period during the 3 months following receipt of an AEHI diagnosis: 1) the need to better understand AEHI and accept one’s status; 2) the need to develop healthy strategies and adjust to the reality of AEHI status; and 3) the need to protect self and others through ART initiation, adherence, safer sex, and safe disclosure. Quotes illustrating these three issues are included in the description below.

### Understanding AEHI and accepting one’s status

During the first interview, participants commented consistently on their emotional reactions to learning about their HIV status, as well as the difficulty in fully absorbing the meaning of AEHI diagnosis. Responses ranged from disbelief or anger to relief. A male patient articulated a sense of denial about his HIV diagnosis and desire to return to his former self:

“My main issue is just the boils [not HIV]. I don’t understand what is happening, this is not my body, the doctor should tell me, help me how to gain back my body. *(21 years old male; first interview)*

A female participant described her initial inability to comprehend and accept her status, as she perceived herself as having minimal risk. She questioned the validity of the diagnosis:

“I was so surprised [with the positive AHI result] and wondered whether the test was genuine. Was it a machine mistake? I didn’t believe it, and told them [healthcare staff] that I need to do a repeat test. There is only one partner I am with… always.” *(25 years old female; first interview)*

Conversely, AHI diagnosis was expected by a sex worker participant due to frequent condomless anal intercourse:

“Okay, in short I am a shoga [gay], we had group sex, we didn’t use protection. I appreciate the way my emotions are, and I accept the way I am. I went into sex work. They gave me ‘a gift’ [HIV]. I felt something was wrong in my body.” *(29 years old male; first interview)*

Despite undergoing lengthy discussions about AHI at the time of diagnosis, participants’ emotional responses to learning about their status may have impeded their ability to comprehend and follow provider recommendations regarding ART initiation and sexual risk reduction. One newly diagnosed woman explained that, despite undergoing a lengthy discussion with her doctor about AHI at the time of diagnosis, she was unable to remember any information about her acute HIV status and its clinical implications.

*“I was not told about [AHI]. I was told, but …if I was told… I didn’t concentrate much. (24 years old female; first interview)*

### Developing healthy strategies and adjusting to the reality of AEHI status

All 13 participants who completed the second interview described the impact of AEHI status on their sex lives. Adjustments, however, depended on participant’s relationship status and social context. Some participants described a period of abstinence following their AEHI diagnosis, while others spoke of the challenge introducing condom use. One young male noted an inability to use condoms with his wife:

“It is difficult to use condoms with your wife because it is a sign of mistrust which is a cause for marital problems.” *(24 years old male; second interview)*

For participants who engaged in sex work, higher payments from customers for having condomless sex often compromised their intentions to practice safer sex.

“…I know many people who do unprotected sex all the time with different partners, and it’s not that they don’t know that condoms are available. Clients offer Ksh 1000 (approx. $10) without condom, claiming that protected sex is not enjoyable…..” *(29 years old male; second interview)*

HIV in coastal families is still associated with behaviours that are not socially accepted. In particular, women who depend economically on their in-laws have a serious dilemma: disclosure could adversely affect their future, while non-disclosure would prevent disclosure benefits. One female participant explicitly asked the counselor to avoid talking about her AEHI diagnosis with her partner and in-laws:

“I cannot confide in her [mother-in-law], she’ll chase me away and keep my child. Promise me, swear by the Mswaff [Quran] to keep it [HIV status] to yourself [counselor]… *(23 years old female; second interview)*

### Protecting self and others through ART initiation, adherence, safer sex, and disclosure

Patients who initiated ART on the day of their diagnosis, reported that being in the acute phase of HIV infection was a major motivating factor for this decision. One 32-year old female participant described during the second interview that she initiated treatment in the context of needing to ‘bring down’ her high viral loads. As such, the participant understood that the significance of receiving her diagnosis during the acute phase was related to how fast she could start treatment. Generally, female participants accepted ART as a way to empower themselves to take continued care of their children. Pregnancy was particularly noted as a motivating factor for starting ART, to avoid onward transmission to the unborn child.

“Yes, I started treatment and I am on treatment. For the baby, I have nevirapine. I sometimes get this feeling that I won’t get to see her grow old.” *(23 years old female; first interview)*

On exploring experiences with immediate treatment, participants reported having high levels of trust in ART and offered testimonies about the effect of ART on restoring and maintaining health. They compared themselves with people they had known who lacked ART access and whose health consequently suffered:

“…unlike earlier where there was no treatment, but now you are helping many people. Some time back you will only be advised about different herbal remedies but now God has been faithful and brought treatment. And if somebody is diagnosed early and starts treatment early they will live normal life”. *(32 years old female; second interview)*

“…it’s a must to use them [ART] as they also help someone do their normal duties. I have accepted to use these drugs because I have been tested and found that I am infected. So, obviously, I have to use them”. *(28 years old female; second interview)*

Participants in their second interview felt more comfortable talking about their experiences. They particularly noted that ART initiation was ‘rewarding’ to recover from AHI symptoms after an initial period of being rather sick.

## Discussion

This paper described general responses and social processes that occurred during the first three months following AEHI diagnosis in a sample of adult males and females in coastal Kenya. Our findings suggested that the period following an AEHI diagnosis involved acquiring basic understanding about the meaning of AEHI status, in addition to undergoing a period of emotional adjustment. Initial responses of confusion, fear, denial, and anger gave way to strategic considerations about partner disclosure and sexual behaviors to reduce risk for onward transmission. At the time of the three-month interview, some participants were still struggling with decisions about disclosure and sexual risk reduction. Barriers to these behavioral adjustments stemmed largely from relationship and family concerns, as well as socioeconomic pressures (e.g. to engage in commercial sex).

Pre-treatment losses to care in AEHI participants in this study were high; 4 of 23 did not register for comprehensive care, and a further 3 patients were lost to follow up shortly after care registration. However, pre-treatment losses in this study correspond with findings of patients newly diagnosed with established HIV infections in other low and lower-middle income countries (Losina et al., 2010; MacPherson, Houben, Glynn, & Kranzer, 2014). Better preparation of potential AEHI patients before AEHI testing is needed, as avoidance coping is a likely explanation for pre-treatment losses to care (Hult, Wrubel, Branstrom, Acree, & Moskowitz, 2012).

Several studies from sub-Saharan Africa show that pregnant women in serodiscordant relationships are particularly vulnerable to HIV-1 acquisition (Brubaker et al., 2011; Nacius, Levison, Minard, Fasser, & Davila, 2013). In our study, two women diagnosed with AEHI were pregnant. This finding highlights the need for all persons, but in particular for pregnant women, to protect self, their unborn baby and partners in order to benefit from HIV prevention and care programming, including immediate ART for those infected and potentially pre-exposure prophylaxis for uninfected partners (Ministry of Health, National AIDS & STI Control Programme, 2016).

More fundamentally, systematic efforts are needed to prepare AEHI patients to cope adequately with the infection, to adopt safer sex strategies, and disclose one’s status. Given the primacy of relationship concerns coupled with high infectivity, couples-focused counseling and active partner notification services are strongly warranted. AEHI participants’ experiences documented in this study call for actionable measures, such as the development of a culturally adapted and manualized counseling intervention to support AEHI patients, specifically addressing the benefits of early ART, and possible transmission interruption (Brenner et al., 2007; Pao et al., 2005; Pilcher et al., 2005; Wawer et al., 2005). Also, quotes from sex worker participants reiterate the need for a sensitive counseling intervention (van der Elst et al., 2013), addressing cultural factors undermining the adoption of condoms in transactional sex.

Findings from this analysis highlight the importance for healthcare providers, especially trained counselors, to take additional time to explore the thoughts, feelings, support systems, and life conditions of newly diagnosed AEHI patients on an ongoing basis, to optimize patients’ motivation and skills to engage in HIV care and adopt safer sex strategies. Additional counseling after the initial diagnosis will likely be necessary to help AEHI patients during this difficult period of transition. In the context of Kenya and elsewhere in sub-Saharan Africa, AEHI counseling should include strategies to better prepare patients and their partners to cope, adjust and sustain behavior changes (Rutstein et al., 2017).

**Figure 1.**
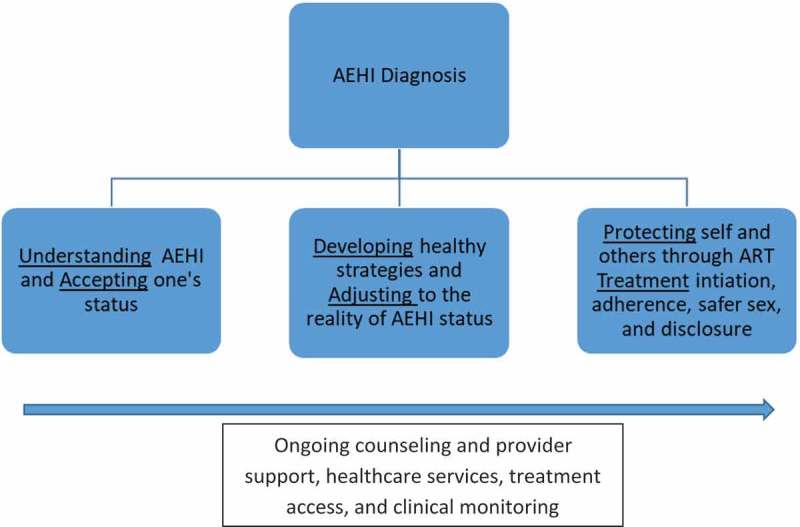
Conceptual model: adjustment to AEHI diagnosis, ART initiation, and sexual risk reduction.

Based on this grounded theory methodology, we offer a preliminary conceptual framework (Figure 1). The framework is meant to guide future research and counseling interventions with patients newly diagnosed with HIV, particularly in the acute and early phases. Although the core concepts of this framework may operate sequentially for many AEHI patients, these concepts may not necessarily follow a systematic temporal or linear trajectory for all patients. Further research is needed to provide empirical support to these core concepts related to AEHI diagnosis, as well as to identify specific mechanisms and provide evidence on how best to facilitate the adjustment needs for each concept in the framework.

There are limitations to this research. First, due to the difficulty in identifying AEHI patients, the sample was small, non-representative, and biased by refusals to enroll in care or participate, as well as attrition of 5 participants after the first interview. Second, our qualitative methods provide only preliminary insights into the key concepts related to AEHI adjustment and ART initiation; further research is needed to validate these concepts and provide detail to guide interventions. Third, despite the data saturation achieved in the interviews, there are other key topics that did not arise in the study which might have important implications for AEHI patients, such as complications of other co-morbid health, long-term ART adherence and patient-provider communication dynamics. Fourth, we did not include a comparison group.

## Conclusion

Given new HIV testing technologies and screening algorithms for diagnosing AEHI, more patients may soon be diagnosed in Kenya and other settings. Our results suggest that additional understanding and systematic intervention may be required to support the needs of patients immediately after receiving, and during the first several months following their AEHI diagnosis, in order to optimize prevention and care outcomes. Findings indicate that social-ecological factors such as culture, traditional gender roles, and economic adversity can determine patients’ ability to communicate with sex partners and respond behaviorally to their AEHI status. However, counseling strategies to promote care engagement and safe disclosure on the same day of HIV diagnosis are critically needed and be sustained over a period of several months to support patients’ adjustment to their diagnosis and reduce the risk of HIV transmission during this important phase of infection.

## References

[CIT0001] Ambrosioni J., Junier T., Delhumeau C., Calmy A., Hirschel B., Zdobnov E., … Yerly S. H. I. V. Cohort Study the Swiss (2012). Impact of highly active antiretroviral therapy on the molecular epidemiology of newly diagnosed HIV infections. *AIDS (London, England)*, 26 16, 2079–2086. 10.1097/QAD.0b013e32835805b6 23052354

[CIT0002] Ananworanich J., Dube K., & Chomont N. (2015). How does the timing of antiretroviral therapy initiation in acute infection affect HIV reservoirs? *Curr Opin HIV AIDS*, 10(1), 18–28. 25415421 10.1097/COH.0000000000000122 PMC4271317

[CIT0003] Brenner B. G., Roger M., Routy J. P., Moisi D., Ntemgwa M., Matte C., … Wainberg M. A. H. I. V. Infection Study Group Quebec Primary (2007). High rates of forward transmission events after acute/early HIV-1 infection. *J Infect Dis*, 195 7, 951–959. 17330784 10.1086/512088

[CIT0004] Brubaker S. G., Bukusi E. A., Odoyo J., Achando J., Okumu A., & Cohen C. R. (2011). Pregnancy and HIV transmission among HIV-discordant couples in a clinical trial in Kisumu, Kenya. *HIV Med*, 12(5), 316–321. 21205129 10.1111/j.1468-1293.2010.00884.x

[CIT0005] Charmaz K. 2006 “Constructing grounded theory: A practical guide through qualitative analysis.”

[CIT0006] Cohen M. S., Shaw G. M., McMichael A. J., & Haynes B. F. (2011). Acute HIV-1 infection. *N Engl J Med*, 364(20), 1943–1954. 21591946 10.1056/NEJMra1011874 PMC3771113

[CIT0007] Cuadros D. F., Awad S. F., & Abu-Raddad L. J. (2013). Mapping HIV clustering: A strategy for identifying populations at high risk of HIV infectin in sub-Saharan Africa. *Int J Health Geogr*, 12(28). Advance online publication. doi:10.1186/1476-072X-12-28 PMC3669110 23692994

[CIT0008] Fiebig E. W., Wright D. J., Rawal B. D., Garrett P. E., Schumacher R. T., Peddada L., … Busch M. P. (2003). Dynamics of HIV viremia and antibody seroconversion in plasma donors: Implications for diagnosis and staging of primary HIV infection. *Aids*, 17(13), 1871–1879. 12960819 10.1097/00002030-200309050-00005

[CIT0009] Ministry of Health, National AIDS & STI Control Programme (2016, July). Guidelines on Use of Antiretroviral Drugs for Treating and Preventing HIV Infection in Kenya 2016. Nairobi, Kenya: NASCOP.

[CIT0010] Hult J. R., Wrubel J., Branstrom R., Acree, M., Moskowitz, J. T(2012). Disclosure and nondisclosure among people newly diagnosed with HIV: An analysis from a stress and coping perspective. *AIDS Patient Care STDS*, 26(3), 181–190. 22256856 10.1089/apc.2011.0282 PMC3286804

[CIT0011] Jones A., Cremin I., Abdullah F., Idoko J., Cherutich P., Kilonzo N., … Dybul M. (2014). Transformation of HIV from pandemic to low-endemic levels: A public health approach to combination prevention. *Lancet*, 384(9939), 272–279. 24740087 10.1016/S0140-6736(13)62230-8

[CIT0012] Krebs S. J., & Ananworanich J. (2016). Immune activation during acute HIV infection and the impact of early antiretroviral therapy. *Curr Opin HIV AIDS*, 11(2), 163–172. 26599167 10.1097/COH.0000000000000228

[CIT0013] Losina E., Bassett I. V., Giddy J., Chetty S., Regan S., Walensky R. P., … Freedberg K. A. (2010). The “ART” of linkage: Pre-treatment loss to care after HIV diagnosis at two PEPFAR sites in Durban, South Africa. *PLosS One*, 5(3), e9538. 10.1371/journal.pone.0009538 PMC2832018 20209059

[CIT0014] MacPherson P., Houben R. M., Glynn J. R., & Kranzer K. (2014). Bull world health organ. *Pre-Treatment Loss to Follow-Up in Tuberculosis Patients in Low- and Lower-Middle Income Countries and High-Burden Countries: a Systematic Review and Meta-Analysis*, 92(2), 126–138. 10.2471/BLT.13.124800 PMC3949536 24623906

[CIT0015] Martin G. E., Gossez M., Williams J. P., Stohr W., Meyerowitz J., Leitman E. M., … Frater J. Spartac Trial Investigators (2017). Post-treatment control or treated controllers? Viral remission in treated and untreated primary HIV infection. *Aids*, 31 4, 477–484. 28060012 10.1097/QAD.0000000000001382 PMC5278888

[CIT0016] Miller W. C., Rosenberg N. E., Rutstein S. E., & Powers K. A. (2010). Role of acute and early HIV infection in the sexual transmission of HIV. *Curr Opin HIV AIDS*, 5(4), 277–282. 20543601 10.1097/COH.0b013e32833a0d3a PMC3130067

[CIT0017] Nacius L. A., Levison J., Minard C. G., Fasser C., & Davila J. A. (2013). Serodiscordance and disclosure among HIV-positive pregnant women in the Southwestern United States. *AIDS Patient Care STDS*, 27(4), 242–247. 23565927 10.1089/apc.2012.0416

[CIT0018] NASCOP 2014 “Guidelines on Use of Antiretroviral Drugs for Treating and Preventing HIVInfection: A rapid advice

[CIT0019] Pao D., Fisher M., Hue S., Dean G., Murphy G., Cane P. A., … Pillay D. (2005). Transmission of HIV-1 during primary infection: Relationship to sexual risk and sexually transmitted infections. *Aids*, 19(1), 85–90. 15627037 10.1097/00002030-200501030-00010

[CIT0020] Pilcher C. D., Fiscus S. A., Nguyen T. Q., Foust E., Wolf L., Williams D., … Leone P. A. (2005). Detection of acute infections during HIV testing in North Carolina. *N Engl J Med*, 352(18), 1873–1883. 15872202 10.1056/NEJMoa042291

[CIT0021] Powers K. A., Ghani A. C., Miller W. C., Hoffman I. F., Pettifor A. E., Kamanga G., … Cohen M. S. (2011). The role of acute and early HIV infection in the spread of HIV and implications for transmission prevention strategies in Lilongwe, Malawi: A modelling study. *Lancet*, 378(9787), 256–268. 21684591 10.1016/S0140-6736(11)60842-8 PMC3274419

[CIT0022] Powers K. A., Miller W. C., Pilcher C. D., Mapanje C., Martinson F. E., Fiscus S. A., … Cohen M. S. U. N. C. Project Acute H. I. V. Study Team Malawi (2007). Improved detection of acute HIV-1 infection in sub-Saharan Africa: Development of a risk score algorithm. *Aids*, 21 16, 2237–2242. 18090052 10.1097/QAD.0b013e3282f08b4d PMC2673577

[CIT0023] Remien R. H., Correale J. A., Bauermeister J., Dubrow J., Bradley M., Steward W. T., … Morin S. F. (2009). Lack of understanding of acute HIV infection among newly-infected persons-implications for prevention and public health: The NIMH multisite acute HIV infection study: II. *AIDS and Behavior*, 13(6), 1046–1053. 19533323 10.1007/s10461-009-9581-7 PMC2787764

[CIT0024] Rutstein S. E., Ananworanich J., Fidler S., Johnson C., Sanders E. J., Sued O., … Tucker J. D. (2017). Clinical and public health implications of acute and early HIV detection and treatment: A scoping review. *Journal of the International AIDS Society*, 20(1), 1–13. 10.7448/IAS.20.1.21579 PMC5515019 28691435

[CIT0025] Sanders E. J., Chirro O., Oduor C., Mangi J., Wahome E., Price M. A., … Graham S. M. 2017 “Outcomes of AHI Screening and Immediate ART Initiation in coastal Kenya.” Conference on Retroviruses and Opportunistic Infections (CROI) Seattle, Washington, USA.

[CIT0026] Sanders E. J., Mugo P., Prins H. A., Wahome E., Thiong’o A. N., Mwashigadi G., … Graham S. M. (2014). Acute HIV-1 infection is as common as malaria in young febrile adults seeking care in coastal Kenya. *Aids*, 28(9), 1357–1363. 24556872 10.1097/QAD.0000000000000245 PMC4032215

[CIT0027] Sanders E. J., Wahome E., Mwangome M., Thiong’o A. N., Okuku H. S., Price M. A., … Graham S. M. (2011). Most adults seek urgent healthcare when acquiring HIV-1 and are frequently treated for malaria in coastal Kenya. *Aids*, 25(9), 1219–1224. 21505300 10.1097/QAD.0b013e3283474ed5

[CIT0028] Sanders E. J., Powers K. A., Werner L., Fegan G., Lavreys L., Mapanje, C., … Graham, S. M. (2015). Targeted screening of at-risk adults for acute HIV-1 infection in sub-Saharan Africa. *Aids*, 29(Suppl 3), 221–230. 26562811 10.1097/QAD.0000000000000924 PMC4714928

[CIT0029] Sereti I., Krebs S. J., Phanuphak N., Fletcher J. L., Slike B., Pinyakorn S., … Utay N. S. (2017). Persistent, albeit reduced, chronic inflammation in persons starting antiretroviral therapy in acute HIV infection. *Clinical Infectious Diseases*, 64(2), 124–131. 27737952 10.1093/cid/ciw683 PMC5215214

[CIT0030] Smith M. K., Rutstein S. E., Powers K. A., Fidler S., Miller W. C., Eron J. J. Jr., & Cohen M. S. (2013). The detection and management of early HIV infection: A clinical and public health emergency. *J Acquir Immune Defic Syndr*, 63(Suppl 2), S187–99. 23764635 10.1097/QAI.0b013e31829871e0 PMC4015137

[CIT0031] van der Elst E. M., Gichuru E., Omar A., Kanungi J., Duby Z., Midoun M., … Operario D. (2013). Experiences of Kenyan healthcare workers providing services to men who have sex with men: Qualitative findings from a sensitivity training programme. *Journal of the International AIDS Society*, 16(Suppl 3), 18741. 24321109 10.7448/IAS.16.4.18741 PMC3852126

[CIT0032] Wawer M. J., Gray R. H., Sewankambo N. K., Serwadda D., Li X., Laeyendecker O., … Quinn T. C. (2005). Rates of HIV-1 transmission per coital act, by stage of HIV-1 infection, in Rakai, Uganda. *J Infect Dis*, 191(9), 1403–1409. 15809897 10.1086/429411

[CIT0033] WHO (2018, November 15). Guideline on when to start antiretroviral therapy and on pre-exposure prophylaxis for HIV. Retrieved from http://apps.who.int/iris/bitstream/10665/186275/1/9789241509565_eng.pdf 26598776

[CIT0034] Zhang X., Zhong L., Romero-Severson E., Alam S. J., Henry C. J., Volz E. M., & Koopman J. S. (2012). Episodic HIV risk behavior can greatly amplify HIV prevalence and the fraction of transmissions from acute HIV infection. *Statistical Communications in Infectious Diseases*, 4(1). 10.1515/1948-4690.1041 PMC3778933 24058722

